# Evaluation of retention of TiTach versus ball and socket attachments used in mandibular implant-retained overdentures: a comparative in vitro study

**DOI:** 10.1186/s12903-025-07056-1

**Published:** 2026-01-30

**Authors:** Nermeen Abdelsalam Rady, Mahmoud Ahmed Atef, Kenda I. Hanno

**Affiliations:** 1 Department of Prosthodontics, Faculty of Dentistry, University of Alexandria, Champolion St, Alexandria, Egypt; 2 Faculty of Dentistry, Alexandria University, Alexandria, Egypt

**Keywords:** Implant overdenture, TiTach attachment, Ball and socket attachment, Retention, Cyclic loading

## Abstract

**Background:**

For the edentulous mandible, removable prostheses that are held in place by implants are great solutions because they offer good retention with various attachments. A novel attachment system that supports an alternative kind of connection is called TiTach. Few studies have investigated this type of attachment. This study compared the retention of TiTach attachments with ball and socket attachments used for overdentures retained by mandibular implants.

**Methods:**

Two ready-made epoxy models were used representing the edentulous mandible and two implants at the canine site with zero inclination were installed. Eight pairs of attachments were used for each model. For the first group, ball and socket attachments were used while for the second group, TiTach attachments were used. Cyclic loading with 1000 insertion and removal cycles was done. Retentive force prior to and following cycles of insertion and removal was measured using the universal testing machine. The Mann Whitney U test and the independent t test were used to compare the groups. Differences in retention values before and after cyclic loading were analyzed using paired t test.

**Results:**

The TiTach group displayed significantly higher retentive force values before cyclic loading (41.68 ± 6.19) and after cyclic loading (30.21 ± 6.78) compared with the ball and socket group before cyclic loading (5.44 ± 0.84) and after cyclic loading (3.20 ± 0.54), respectively (*P* < 0.0001). Both groups exhibited a statistically significant decline in retentive force after cyclic loading (*P* < 0.0001). Retention loss in the ball and socket group is 2.35 (from 5.41 to 3.06), representing a 43.44% decrease, while retention loss in the TiTach group is 10.30 (from 40.90 to 30.60), representing a 25.18% decrease.

**Conclusion:**

Higher initial and final retentive force as well as a beneficial lower percentage change in retentive force were demonstrated by the TiTach attachment compared with the ball and socket attachment. The TiTach attachment could be a suitable choice when increased retention is required.

**Supplementary Information:**

The online version contains supplementary material available at 10.1186/s12903-025-07056-1.

## Background

Despite a decline in total tooth loss over the past decade, edentulism remains a pressing global health concern, especially for elderly people, with far-reaching consequences for their well-being [1, 2].

The use of implant-retained prostheses, which preserve bone, is recommended by Carlsson [3] as an alternative to the ongoing bone resorption that occurs under a complete denture.

When the effects of implant overdentures and conventional dentures on the resorption of the posterior mandibular residual ridge were compared over a 5-year period, the conventional denture group experienced a mean decrease in alveolar height of 1.63 mm, whereas the implant overdenture group experienced a reduction of 0.69 mm [4].

The 2009 York Consensus recommended that the minimum used as a first line of treatment for edentulous patients is a mandibular overdenture supported by two implants. A significant perceived obstacle to implant-supported prosthesis is the expense. The expense of this treatment approach is justified by supporters throughout the course of the patient’s life [5]. The implant supported prostheses are certainly more expensive than conventional dentures but the use of just two implants can keep the initial cost to a minimum. Furthermore, deferring the whole cost over the expected life span of the patient shows that the annual difference in costs between the two modalities is relatively small, especially compared with the initial first year costs.

For mandibular implant overdentures, different overdenture attachment systems can be employed to improve denture stability and retention. The most often used attachment methods are the ball, bar, and magnet varieties as well as several separate mechanical attachments that are sized and designed similarly to ball forms [6].

The most often used overdentures use a ball-shaped retaining system. Its benefits include a straightforward manufacturing process, a large range of mobility, affordability, ease of use and maintenance, good retention, maintenance of cleanliness, and high patient satisfaction [7]. But because the ball attachment abutment requires implants to be positioned parallel, losing parallelism might make it more difficult to insert and remove the prosthesis or break the abutment. Furthermore, because the O-ring is prone to wear, it must be replaced on a regular basis [7].

Unlike the nylon attachment methods, a novel attachment mechanism permits metal-to-metal contact between the abutment and its cap. The silicone sleeve, TiTach cap, and TiTach abutment make up the three components of the TiTach attachment. The metal cap features vertical grooves that allow it to open when the abutment is engaged. The sleeve made of silicone serves as a block-out component while the cap is being included. The silicone sleeve is cut into two halves after the pick-up procedure and placed between the housing and the cap, where it latches beneath the outside edge of the cap [8].

The TiTach prosthetic system was created to address the issue of overdentures with implant assistance. It can be used to indications that call for 66 degrees of divergence between contralateral implants or up to 33 degrees of divergence for a single implant. To fit the cap, it needs a 6 mm diameter and 4.5 mm of vertical clearance [9].

Up to 0.2 mm of vertical cushioning is permitted, allowing for progressive prosthesis seating and mucosal compression during function and parafunction. Furthermore, every attachment has a force resistance of seven to ten pounds [9]. However, achieving optimal alignment and seating of the TiTach components may require precise implant placement and clinical expertise. Misalignment can compromise retention, patient comfort and wear characteristics.

The effectiveness of overdenture depends on retentive power. When selecting the proper attachment method for an overdenture, it is imperative to take into account the biomaterial features of the attachment [10].

Important considerations for choosing attachments for an implant-retained overdenture include the retentive tension and strain absorbed during removal. Studies evaluating the TiTach attachment are limited in the literature [8, 9, 11, 12]. A study comparing TiTach with locator attachments concluded that TiTach attachments showed less stable retention over time compared to locator attachments [11], and increased wear after 2 years of simulated clinical use [12]. Although multiple studies have evaluated the performance of TITACH compared to locator attachments, limited evidence exists comparing TITACH with ball and socket systems. Given the continued clinical use of ball and socket attachments and their distinct mechanical properties, a direct comparison is needed to guide attachment selection based on clinical performance, patient satisfaction, and maintenance requirements.

Therefore, the purpose of this study is to analyze and contrast the retentive force values of ball and socket and TiTach attachments used for implant-retained mandibular overdentures before and after cyclic loading.

The null hypotheses are that there is no difference in retentive force values between ball and socket and TiTach attachments and that cyclic loading will have no effect on the retentive forces in both groups.

## Methods

The Committee of Research Ethics in Alexandria University, Faculty of Dentistry (IORG 0008839) with a number of 0894–03/2024 has provided approval of the research before any activities related to the research.

The sample size was determined using 80% research power and a 5% alpha error assumption. The mean ± SD retention values were reported to be 61.50 ± 5.94 for TiTach attachments [11], while it was 50.48 ± 4.83 for the ball attachments [12]. Based on comparison between independent means using the highest SD = 5.94 to ensure enough study power. The sample was calculated to be 6 samples per group yielding an effect size of 1.855 and this was increased to 8 samples to compensate for processing errors. Total sample = Number of samples per group x Number of groups = 8 × 2 = 16 samples. Sample size was based on Rosner’s method [13] calculated by G*Power 3.1.9.7 [14].

Two completely edentulous mandibular models, fabricated from 7.5 mm wide epoxy resin in the canine region [15], were utilized (Ramses Medical Products Factory). This resin was overlaid with a 1.5 mm thick flexible polyurethane that simulates oral mucosa. Subsequently, 16 stone casts were produced from the epoxy model. On these casts, anterior and posterior acrylic resin teeth (Acrostone Plus; Acrostone Co Ltd) were positioned on bases for mandibular trial dentures with wax occlusion rims. Similar to the natural distance between the two canines, the mandibular arch’s intercanine distance was 22 mm, or 11 mm from the midline [16].

Heat-polymerizing resin (SR Triplex Hot; Ivoclar Vivadent), was used to process 16 mandibular trial dentures, then finished and polished conventionally. A surgical guide printed from a clear photoinitiated resin (Surgical guide resin; Formlabs) was used. The guide was designed using a software program (Bluesky plan; Bluesky) and produced via 3D printing (Formlabs 2; Formlabs) to ensure accurate drilling of implants in the bilateral canine region. Following a specific drilling sequence, 2 implants (Implanova; Dental Evolutions Inc) were installed parallel to each other, each with a length of 10 mm and a diameter of 3.5 mm, achieving a primary stability of 35 Ncm using a torque wrench.

**Fig. 1:**
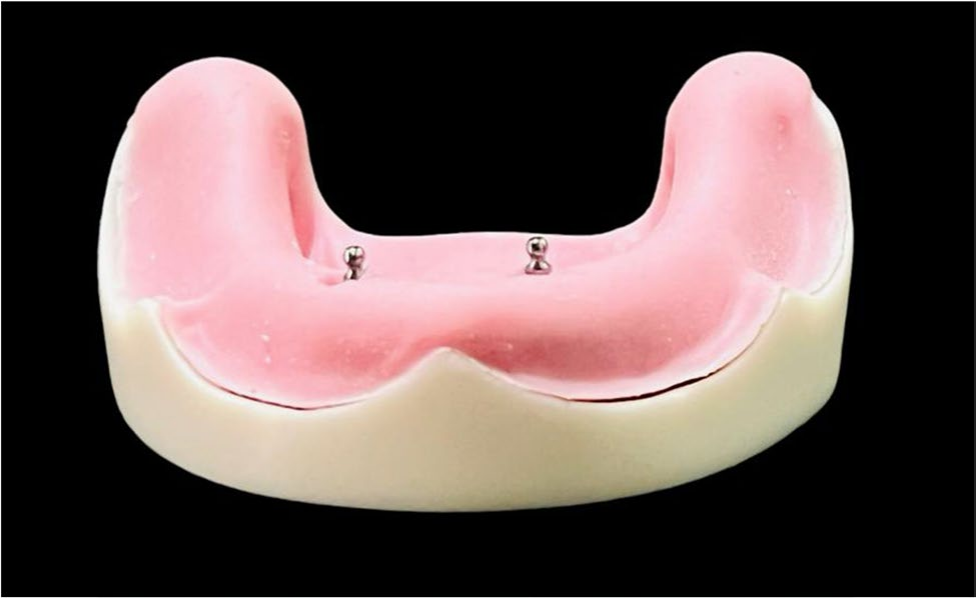
Ball abutments placed in position over epoxy model

**Fig. 2:**
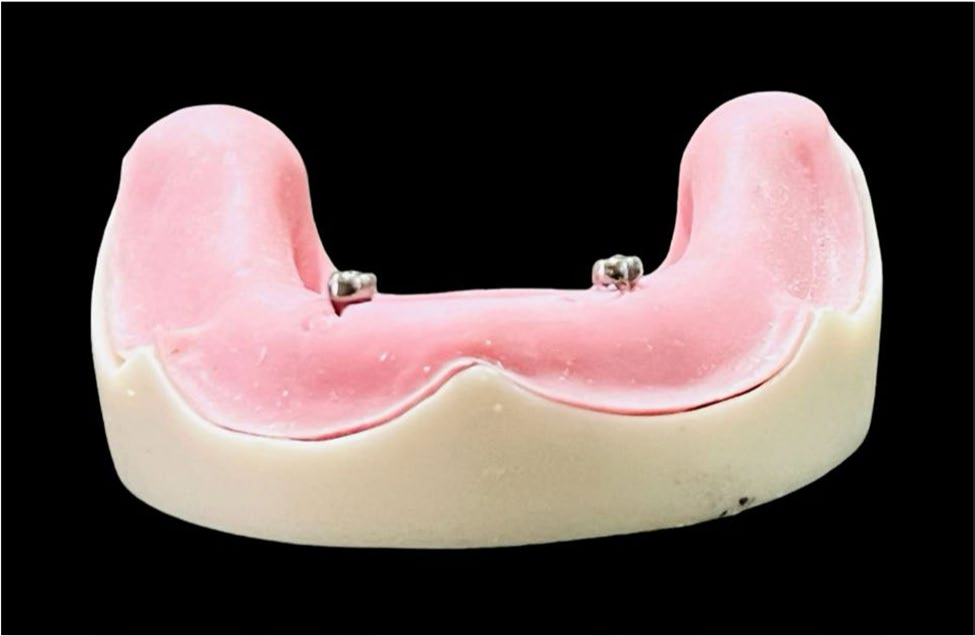
TiTach placed in position over the epoxy model

Two attachment systems, ball and socket Attachment (Neobiotech Inc) and TiTach (Dental Evolutions Inc), were employed. Attachments were screwed according to each group. The abutments for the ball and socket (Fig. 1) and TiTach (Fig. 2) were attached to the implant with a torque of 20 Ncm using a torque wrench following the manufacturer’s instructions.

For the ball and socket group, the attachment housing was positioned over the abutment until a definite position was established. The overdenture was then seated over the cast, and the attachment positions were marked and relieved to ensure proper seating. Additionally, lingual vent holes were incorporated in the overdenture to allow excess resin escape.

**Fig. 3:**
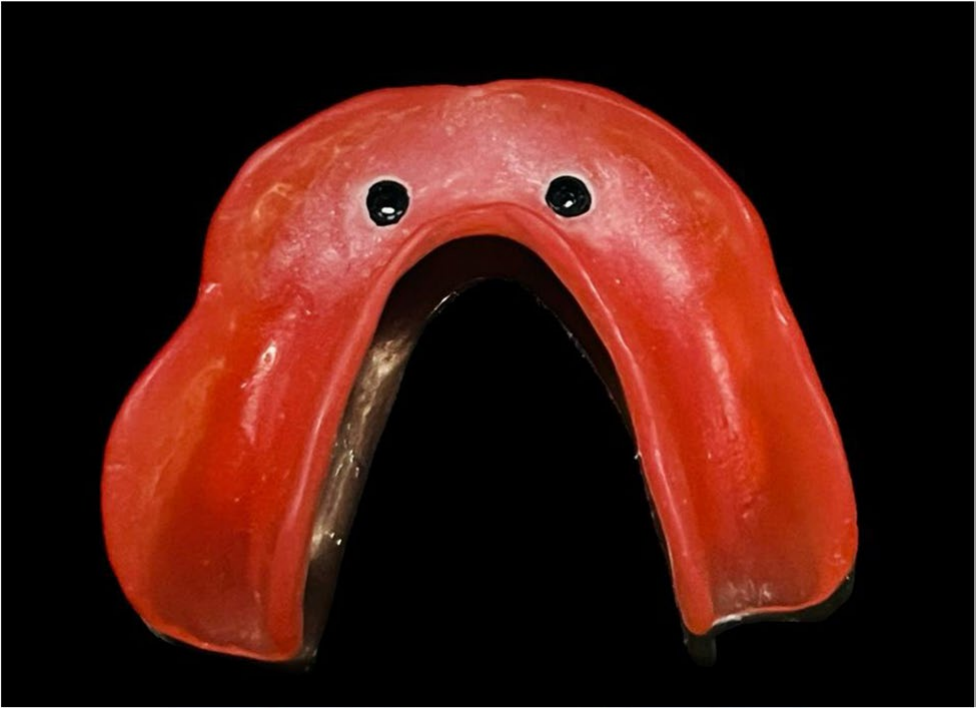
Overdenture with ball and socket attachment after pick up

The process involved mixing autopolymerizing resin (Cold cure resin, Acrostone Co Ltd) and applying monomer in the vent openings. Once the dough stage of the acrylic resin had been reached, it was inserted into the overdenture’s fitting surface and positioned over the model to retrieve the attachment caps. Following this, the overdenture was detached from the abutments, and finishing and polishing was accomplished. The male black processing component was then replaced with a clear socket nylon insert using an insert tool (Fig. 3). Each denture of the 8 dentures in this group was picked up from the epoxy resin model using the same method.

**Fig. 4:**
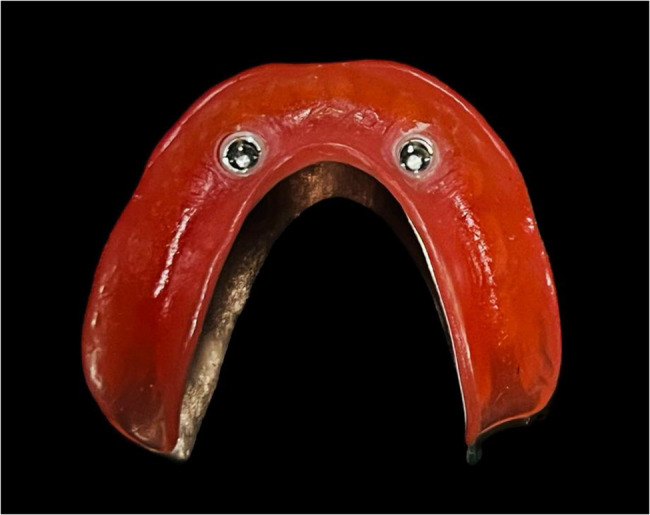
Overdenture with TiTach attachment caps after pick up

For TiTach group, when the TiTach silicone sleeve was put on the cap, care was taken to ensure that the retentive fins were not covered, and that the sleeve’s top was beneath the cap’s upper border. The assembly of the cap and sleeve was secured on top of the abutment until it was in a firm position. The sleeve prevented locking of the acrylic resin in the abutment by covering the whole abutment neck that protruded from the gingival area. The autopolymerizing resin was mixed and same procedure completed in the ball and socket group was repeated for the TiTach group (Fig. 4). Following the curing of the resin, the silicone sleeve was taken off, and a scalpel blade was used to cut the protruding portion in half. Each denture of the 8 dentures in this group was picked up from the epoxy resin model using the same method.

### Retention measurement

**Fig. 5:**
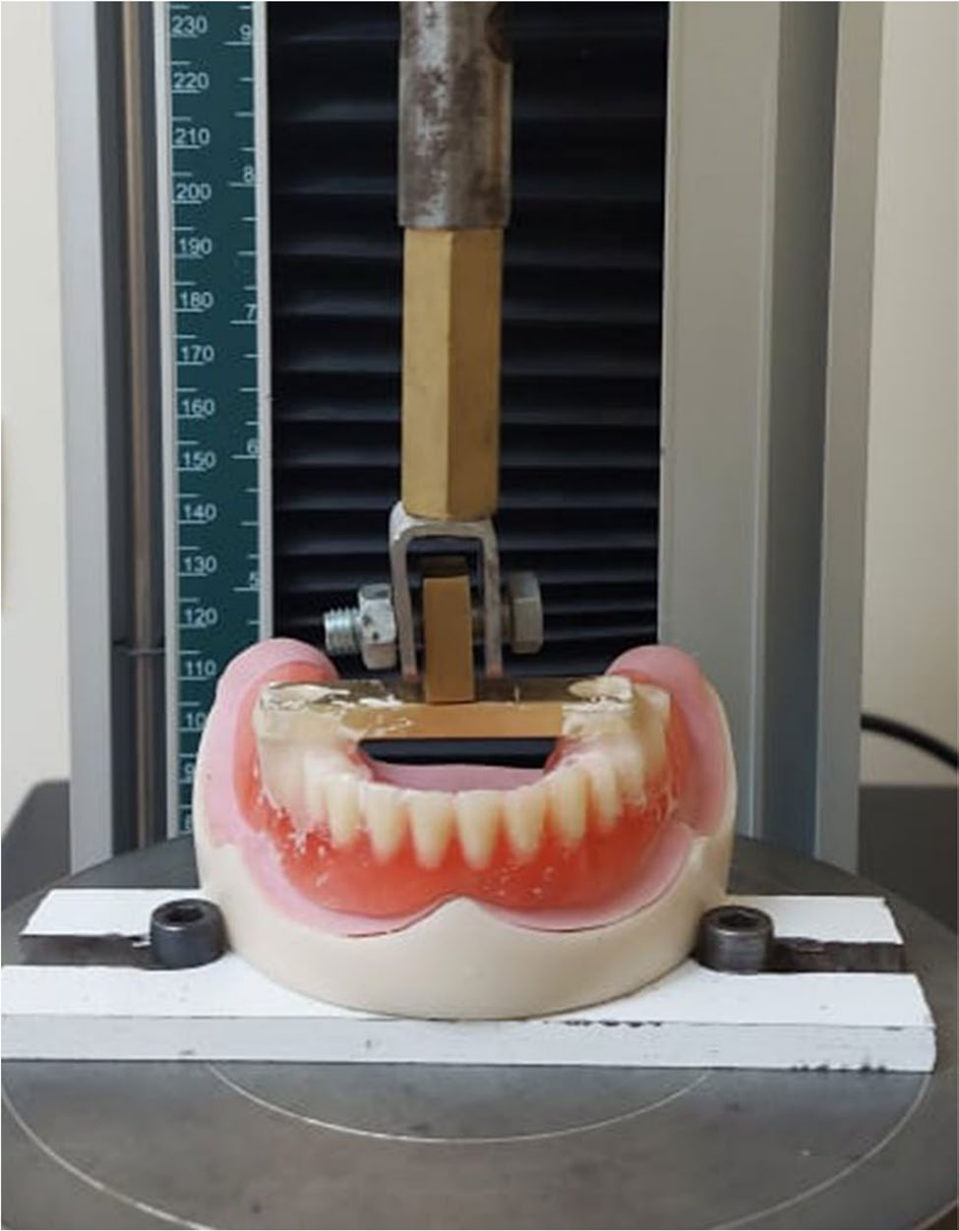
Overdenture dislodgment by using universal testing machine

A universal testing device (Multi Test5-XT; Mecmesin Corp) was used to remove the overdenture (Fig. 5). The 90-degree customised jig was used to secure the model to the lower part of the universal testing device. The jig was designed to ensure accurate and repeatable positioning of the specimen during testing. The jig was customized to fit the model and ensure a 90-degree angle. A specially designed T-shaped metal plate with a vertical part was affixed to the universal testing machine’s upper member to give the overdentures a tensile dislodging force [17].

Using autopolymerizing resin (Acrostone Co. Ltd.), the metal plate was affixed to the acrylic resin teeth’s occlusal surfaces in the first molar areas. This made sure that the model and the metal plate were parallel. To replicate the axial dislodging forces of a denture, a tensile force perpendicular to the occlusal plane was applied as feasible.

The universal testing machine’s crosshead speed was adjusted at 50 mm/min to withdraw the overdenture, simulating the rate at which a prosthesis would separate from the remaining alveolar ridge during masticating and up to a 4 mm extension [18].

**Fig. 6:**
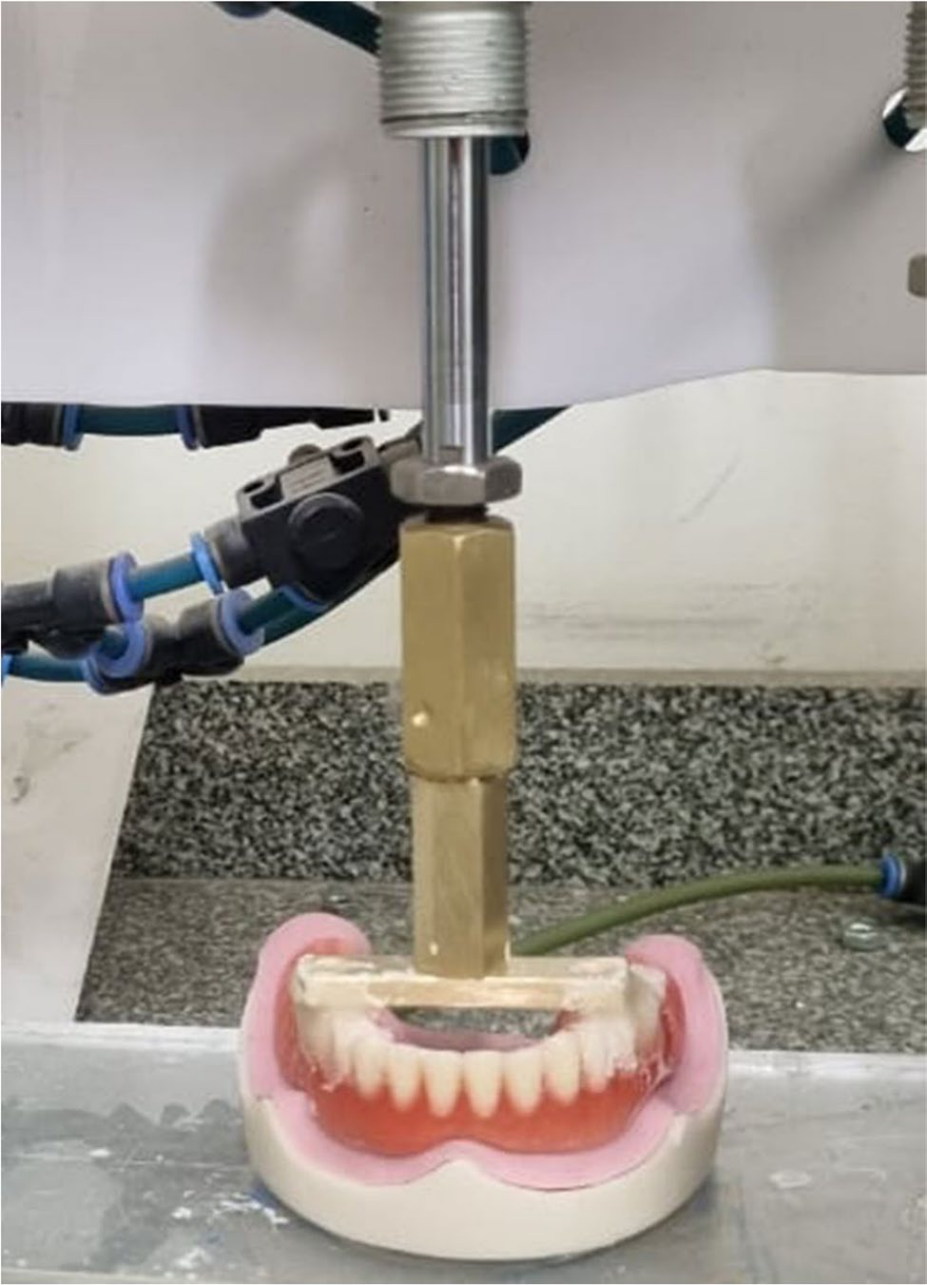
Overdenture subjected to 1000 cycles in cyclic loading machine

A custom-made cyclic loading machine that served as a simulator for mastication was used to replicate insertion and removal of the overdentures in order to conduct a vertically oriented cyclic tension-compression test. Based on 3 removal-insertion cycles per day on average, each overdenture was put through 1000 cycles, which is equivalent to the typical annual number of cycles of insertion and removal [19] (Fig. 6).

The peak load to dislodgement was recorded in order to determine the final retentive force of each attachment system. Peak load is the maximum amount of force applied to the attachment system before it dislodges.

The force needed to remove the overdentures was measured both before and after the tension-compression cycles. The difference that resulted showed that usage has resulted in decreased retention [20].

To confirm normality, Q-Q plots and the Shapiro-Wilk test were employed. Because the retention values were normally distributed, the data was mostly presented using the mean, standard deviation (SD), and 95% confidence interval (CI). Since retention loss was not normally distributed, median, minimum, and maximum values were mostly employed for data presentation. Retention loss was estimated using the method [values following cyclic loading – values before cyclic loading)/values before cyclic loading] x 100. The Mann Whitney U test and the independent t test were used to contrast the groups. The paired t test was used for comparing retention values prior to and following cyclic loading. The level of significance was established at *P* value ≤ 0.05, and all tests were two-tailed. IBM SPSS, version 23 for Windows, Armonk, NY, USA, was used for analysis of the data.

## Results

Table 1 shows a comparison of the retention values of both ball and socket and TiTach attachments before and after cyclic loading. The TiTach group displayed significantly (*P* < 0.0001) higher retentive force values before cyclic loading (41.68 ± 6.19) and after cyclic loading (30.21 ± 6.78) compared with the Ball and socket group (5.44 ± 0.84 and 3.20 ± 0.54, respectively. There was a statistically significant decline in retentive force in both groups after cyclic loading (*P* < 0.0001).

**Table 1:** Retention values (Newtons) of ball and socket and TiTach attachments before and after cyclic loading

Cyclic loading		TiTach (*n* = 8)	Ball and socket (*n* = 8)	*P* value
Before	Mean ± SD	41.68 ± 6.19	5.44 ± 0.84	< 0.0001*
	95% CI	36.92, 46.44	4.80, 6.09	
	Median	40.90	5.41	
	Min - Max	33.70–51.60	4.39–6.88	
After	Mean ± SD	30.21 ± 6.78	3.20 ± 0.54	< 0.0001*
	95% CI	25.00, 35.42	2.79, 3.61	
	Median	30.60	3.06	
	Min - Max	22.10–41.50	2.28–3.89	
*P* value		< 0.0001*	< 0.0001*	

*Statistically significant difference at *P* ≤ 0.05

Table 2 shows retention loss of ball and socket and TiTach attachments after cyclic loading. The mean values of retention loss of ball and socket and TiTach attachments after cyclic loading were 40.38 ± 11.35 and 28.18 ± 6.08 respectively. For the Ball and socket system, retention loss is 2.35 (from 5.41 to 3.06), representing a 43.44% decrease. For the TiTach system, retention loss is 10.30 (from 40.90 to 30.60), representing a 25.18% decrease. Statistical significance was found between the retention loss of ball and socket and TiTach attachments after cyclic loading (*P* = 0.019). Ball and socket attachments showed significantly greater values of retention loss compared to TiTach attachments.

**Table 2:** Retention loss (in %) of ball and socket and TiTach attachments after cyclic loading

Cyclic loading		Ball and socket (*n* = 8)	TiTach (*n* = 8)	*P* value
After	Mean ± SD	40.38 ± 11.35	28.18 ± 6.08	0.019*
	95% CI	31.66, 49.11	23.51, 32.85	
	Median	42.26	28.66	
	Min - Max	19.13–58.24	19.57–37.57	

*Statistically significant difference at *P* ≤ 0.05

## Discussion

The present study compared the retention of the ball and socket and TiTach attachments in mandibular implant-retained overdentures regarding before and after cyclic loading. The null hypotheses were disproven, as the retentive force values of ball and socket and TiTach attachments differed statistically significantly, and cyclic loading had a significant effect on the retentive forces in both groups.

Epoxy resin was chosen for implant installation due to its suitable modulus of elasticity similar to bone (around 20 GPa), ease of machining, and ample toughness for cyclic testing [15] 1.

The teeth were positioned conventionally in the mandibular trial denture, with the canines set at a distance of 22 mm away from each other (11 mm measured from the central line), resembling the natural canine distance [16]. In accordance with the McGill and York consensus advice that a two-implant overdenture is the main therapy for the edentulous mandible, 2 implants were inserted in the epoxy model [16].

The study chose to assess the TiTach attachment since it is a novel attachment featuring a specific metal-to-metal interface between the abutment and its cap. For comparison, the ball and socket attachments were used to compare with TiTach attachment to check retention for implant retained overdenture.

A specially designed 90-degree jig was used to secure the model to the bottom portion of the universal testing device in order to measure the retentive force of the overdentures under investigation. This allowed for the application of tensile force perpendicular to the occlusal plane in order to simulate axially directed dislodging forces during denture function [11].

In order to minimize measurement mistakes caused by uncontrolled variations in slack while employing several chains to link to the testing apparatus, a metal plate that is T-shaped was also used to establish application of load at the mandibular arch’s center [21].

To simulate the speed at which a prosthesis would separate from the remaining alveolar ridge during chewing, the universal testing machine’s crosshead speed was set at 50 mm/min up to a 4 mm extension, which corresponds to the two attachments’ vertical heights (4.50 mm) [18, 22]. Each attachment system’s initial and ultimate retentive forces were captured by the computer attached to the universal testing equipment, which also recorded the peak load to dislodgement [21].

To mimic the insertion and removal of the 16 overdentures, a cyclic loading machine was employed in a vertical cyclic tension-compression test. Considering 3 removal-insertion cycles on average each day, each overdenture endured 1000 cycles, which represents the mean number of insertion and removal cycles in a year [19].

Our results revealed that there was a statistically significant difference between both attachments where TiTach attachment recorded higher values of retentive forces than ball and socket attachment both before and after cyclic loading.

The results are in line with Ramadan et al. [11], who investigated the retention of mandibular overdentures retained with locator and TiTach attachments on implants before and after 1000 insertion and removal cycles and reported that TiTach attachments had higher final retentive force values after wear simulation.

This could be attributed to the design of the TiTach attachments. The metal cap of the attachment had several metallic lamellae that engage the circumferential undercut of the TiTach abutments. The rigidity of these lamellae appears to maintain the retention forces even after wear and minimize the need of metal cap reactivation [11].

The results of the present study showed that cyclic loading significantly decreased retentive force values in both groups. However, the percentage loss of retention in ball and socket attachment was significantly higher than TiTach attachment. The decreased retention values with time, was in the agreement with Hassan RN et al. [23] and Elsonbaty et al. [12] who reported that surface alterations in the nylon components under cyclic loading can be used to explain this retention loss. Additionally, attachment systems wear down while in use, which results in a reduction or even loss of retention [24].

Parallel to our results, Doukas et al. [25] found that after six months of repeated manual removals, there was a notable drop in retention in ball attachments with titanium ball patrix and noble alloy matrix, ranging from 32% to 50% depending on the inter-implant distance.

Our results are also consistent with Eladrosi et al. [8] who investigated retentive forces of direct and indirect incorporation of TiTach attachment at different evaluation time. They concluded that statistically significant reduction in retention force was found after 6 months of overdenture insertion, however the values were still clinically acceptable.

El Khourazaty NS [26] compared between ball and socket and Polyether ether ketone (PEEK) attachment, highlighted that no statistically significant variations in retention were observed between the two attachments at the time of denture insertion. The highest retention was found in ball and socket attachment. However, after six months, comparing the retention values of the two attachments, PEEK attachments showed higher retention values compared with ball and socket attachments. They concluded that PEEK attachment outperforms traditional ball and socket attachment with nylon matrix in terms of retention values [26].

Shastry et al. [27] found that bar and clip attachment demonstrated the highest retentive force, followed by locator attachment, and finally the ball/O-ring attachment. After putting the three attachment systems through temperature cycles, a statistically significant drop in retention force was seen [27]. These results coincide with our results because the ball and socket attachment exhibited much lower retention values. The initial retentive force decreased as a result of ball attachment component deterioration by 32–50% [27].

According to Passia et al. [28] and Ludwig et al. [29] because of friction between male and female parts, resilient attachments wear under functional loading or after several cycles of insertion and removal.

Numerous authors conclude that the ball attachment is the most often utilized attachment for un-splinted implants because it is practical, affordable, and efficient [30]. The usage of ball attachment was also found to be beneficial in terms of reducing denture movement when comparing stability in mandibular implant retained overdentures with bar, magnet, or ball attachment [31].

According to research, the majority of the attachment systems exhibited a general tendency toward a decrease or complete loss of retentive forces. Additionally, a slow and ongoing loss of ball and socket attachment retention resulted from repeated insertion-removal cycles. As a result, the clinician should repeat the evaluation every year or every two years [31].

Retentive force can be affected by various factors, including the temperature, implant inclination, oral habits that are parafunctional, salivary presence, and distance between implants. This in vitro study had some limitations, such as that the overdenture did not represent the patient’s parafunctional oral habits, and masticatory function. Other restrictions include the lack of temperature variations, as well as the dry testing environment devoid of saliva. One notable limitation of this study is the use of an epoxy resin model rather than actual bone to simulate implant placement. While epoxy resin offers consistency, ease of manipulation, and standardized mechanical properties, it does not replicate the heterogeneous nature of human bone. Natural bone exhibits variable cortical and trabecular densities, which influence implant stability, micromovements, and ultimately, the retention behavior of overdenture attachments under functional loading. Additionally, bone undergoes biological remodeling and adaptive changes in response to mechanical stress—factors that epoxy models cannot simulate. Another limitation of the present study is the use of only vertical cyclic tension-compression testing to evaluate retention behavior. While this loading direction reflects the path of insertion and removal of overdentures, thus offering clinically relevant baseline data, it does not fully replicate the multi-directional forces experienced during mastication and daily function. In the oral environment, non-axial dislodging forces, such as anterior, posterior, and lateral stresses, may influence attachment wear, retention stability, and overall prosthesis performance.

It is advisable to conduct further studies to assess the wear characteristics of the TiTach attachment after use, and additional studies are warranted to evaluate its performance with different inclination of implants. Furthermore, clinical studies should be conducted to explore the impact of utilizing the TiTach attachment on osseointegration of dental implants. Future studies incorporating multi-directional mechanical testing are needed to better simulate intraoral conditions and improve the clinical translatability of the findings. Additionally, the manufacturer of the TiTach attachment should consider producing attachments with a wide variety of collar heights to accommodate a range of thicknesses of the oral mucosa.

## Conclusions

The findings of this in vitro investigation led to the following deductions:

1. In comparison to the ball and socket attachment, the TiTach attachment exhibited a beneficial lower retentive force change as a percentage as well as higher initial and final values of retentive force.
2. The TiTach attachment could be a suitable choice when increased retention is required.

## Supplementary Information

Supplementary Material 1.

## Data Availability

The datasets used and/or analysed during the current study are available from the corresponding author on reasonable request.
