# Supplementary material for: Evaluation of retention of TiTach versus ball and socket attachments used in mandibular implant-retained overdentures: a comparative in vitro study
Source: BMC Oral Health. 2026 Jan 30;26:377. doi: 10.1186/s12903-025-07056-1 (PMC12933911; doi:10.1186/s12903-025-07056-1)
Supplement: Supplementary file 1 — Supplementary Material 1. [file 12903_2025_7056_MOESM1_ESM.docx]

**Figure showing the difference in retention values between the Ball and socket and the Titach group before and after cyclic loading.**
